# Incidentally detected follicular thyroid carcinoma mimicking parathyroid adenoma on Tc-99m MIBI scan: A case report

**DOI:** 10.1097/MD.0000000000038107

**Published:** 2024-05-03

**Authors:** Yeon-Hee Han, Hwan-Jeong Jeong, Sun Young Lee, Seok Tae Lim

**Affiliations:** a Department of Nuclear Medicine, Research Institute of Clinical Medicine of Jeonbuk National University-Biomedical Research Institute of Jeonbuk National University Hospital, Cyclotron Research Center, Molecular Imaging and Therapeutic Medicine Research Center, Jeonbuk National University Medical School and Hospital, Jeonju, Jeonbuk, Republic of Korea; b Department of Radiation Oncology, Research Institute of Clinical Medicine of Jeonbuk National University-Biomedical Research Institute of Jeonbuk National University Hospital, Jeonbuk National University Medical School and Hospital, Jeonju, Jeonbuk, Republic of Korea.

**Keywords:** case report, follicular thyroid carcinoma, parathyroid adenoma, SPECT/CT, Tc-99m MIBI

## Abstract

**Rationale:**

Primary hyperparathyroidism, though relatively prevalent among endocrine disorders, affecting 1% of the general population, often presents diagnostic challenges. Given its potential to precipitate severe complications including nephrolithiasis and fractures, timely diagnosis, and effective management are crucial.

**Patient concerns:**

A 38-year-old woman with hypercalcemia was referred to the Department of Nuclear Medicine for a Tc-99m MIBI scan.

**Diagnoses:**

Tc-99m MIBI scan showed focal increased uptake in the left thyroid gland area, initially suggesting a parathyroid adenoma. Further examination using SPECT/CT revealed a nodular lesion within the left thyroid gland showing high Tc-99m MIBI uptake.

**Interventions:**

Left thyroid lumpectomy confirmed the lesion as follicular thyroid carcinoma. On the second Tc-99m MIBI scan conducted after total thyroidectomy, a parathyroid adenoma was eventually detected in the right lower area, enabling the subsequent appropriate treatment, a right lower parathyroidectomy.

**Outcomes:**

Thirteen days after the parathyroidectomy, serum levels of total calcium and parathyroid hormone returned to normal. Furthermore, bone mineral density evaluated using DEXA remained within the expected range for her age even after 14 months.

**Lessons:**

When interpreting the Tc-99m MIBI scan, it is essential to keep in mind that various tumors rich in mitochondria, such as thyroid carcinoma, could show a high uptake of Tc-99m MIBI.

## 1. Introduction

Primary hyperparathyroidism is a common endocrine disorder, affecting approximately 1% of the overall population.^[1]^ This is characterized by excessive production of parathyroid hormone (PTH) due to an overactive parathyroid gland, leading to a significant increase in serum calcium levels.^[2]^ It is caused by a sporadic parathyroid adenoma (80%–85%), multiglandular parathyroid hyperplasia (15%–20%), or parathyroid carcinoma (<0.5%).^[3]^ Because hyperparathyroidism can lead to adverse complications such as nephrolithiasis and fractures, early diagnosis and appropriate treatment are crucial. Dual-phase Tc-99m methoxyisobutylisonitrile (MIBI) scan is considered the primary functional imaging modality for detecting functional parathyroid adenoma. Due to its significant affinity for mitochondria, Tc-99m MIBI has become the preferred choice for localizing parathyroid adenomas.^[4]^ While Tc-99m MIBI scan remains a valuable tool for detecting parathyroid adenomas, comprehensive knowledge of the diverse conditions exhibiting high Tc-99m MIBI uptake is essential for accurate and informed diagnostic decisions. Over the past decade, a hybrid imaging of single-photon emission computed tomography/computed tomography (SPECT/CT) has been employed in clinical field, providing precise localization of various diseases with high diagnostic accuracy. Tc-99m MIBI SPECT/CT is no exception. It has the advantage of higher sensitivity compared to a planar scan or other radiological images.

Here, we present a case of incidentally detected thyroid carcinoma mimicking parathyroid adenoma on a Tc-99m MIBI scan in a patient with hypercalcemia.

## 2. Case presentation

**Figure 1.**
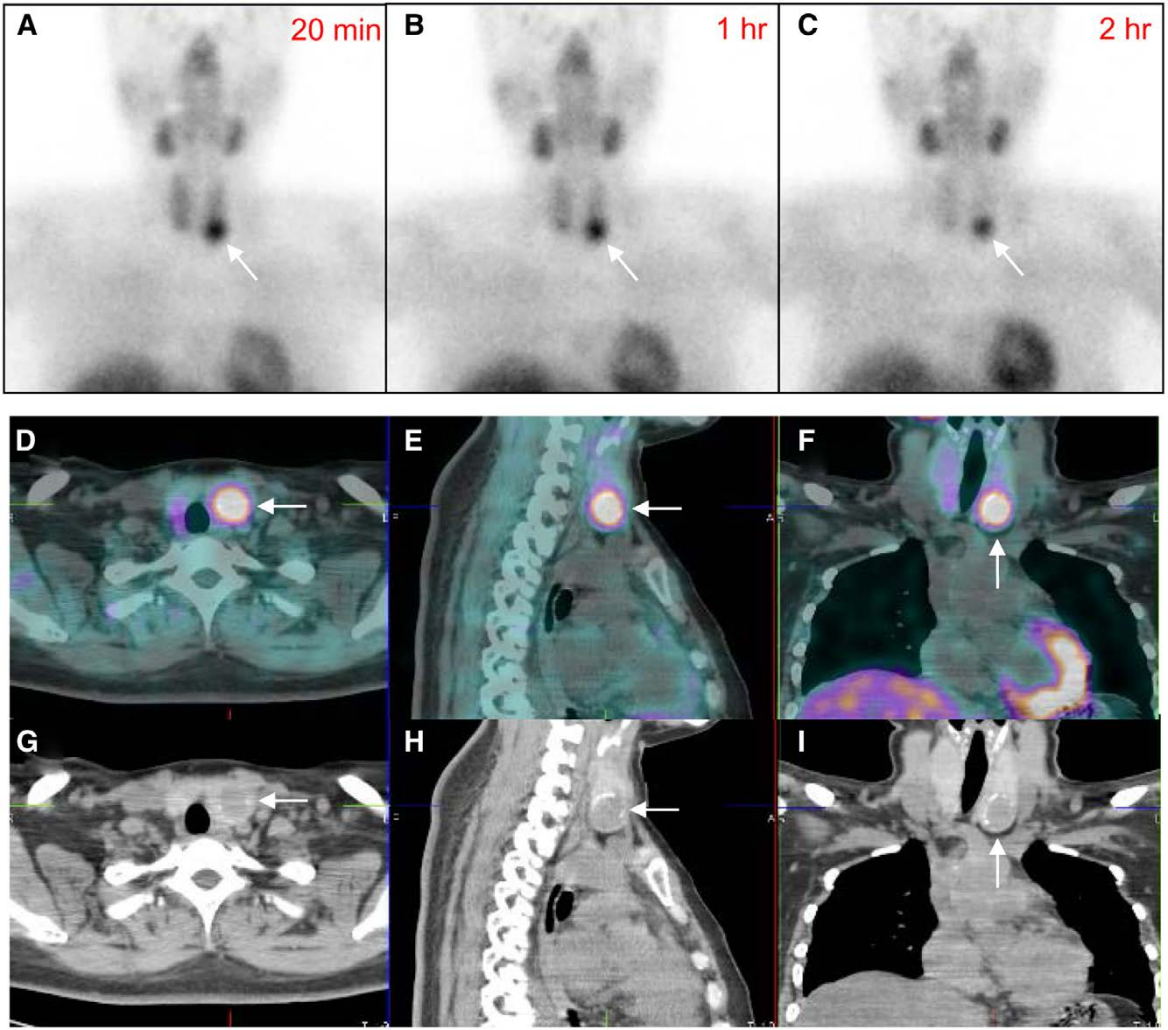
Initial Tc-99m MIBI scan and SPECT/CT. The 20-min image showed focally increased uptake in the left thyroid gland area (A). The uptake continued on the 1-h and 2-h images (B, C). The SPECT/CT revealed a 2.1-cm nodular lesion within the left thyroid gland exhibiting high Tc-99m MIBI uptake (D–F, fusion images, G–I CT images). Arrows indicate the lesion.

A 38-year-old woman was found to have high total calcium levels of 12.1 mg/dL (normal range: 8.6–10.0 mg/dL) and elevated PTH levels of 105.4 (normal range: 11.0–62.0 pg/mL) during a routine health checkup. As part of the investigation for hyperparathyroidism, a Tc-99m MIBI scan was conducted (Fig. 1). The 20-minute image showed focally increased uptake in the lower portion of the left thyroid gland area and it continued on the 1-hour and 2-hour images. A diagnosis of left lower parathyroid adenoma was strongly suspected. To obtain a more accurate evaluation, single-photon emission computed tomography/computed tomography (SPECT/CT) imaging was performed. It revealed a 2.1-cm sized low attenuated nodule with calcifications around its margin. The nodular lesion was located within the left thyroid gland exhibiting high Tc-99m MIBI uptake. Initially, an intrathyroidal parathyroid adenoma was suspected. Subsequently, a left thyroid lumpectomy was performed. Unlike what was expected, the lesion was confirmed as a minimally invasive follicular thyroid carcinoma upon histologic examination. As a result, the patient underwent a total thyroidectomy and, 4 months later, received a high-dose radioiodine therapy.

**Figure 2.**
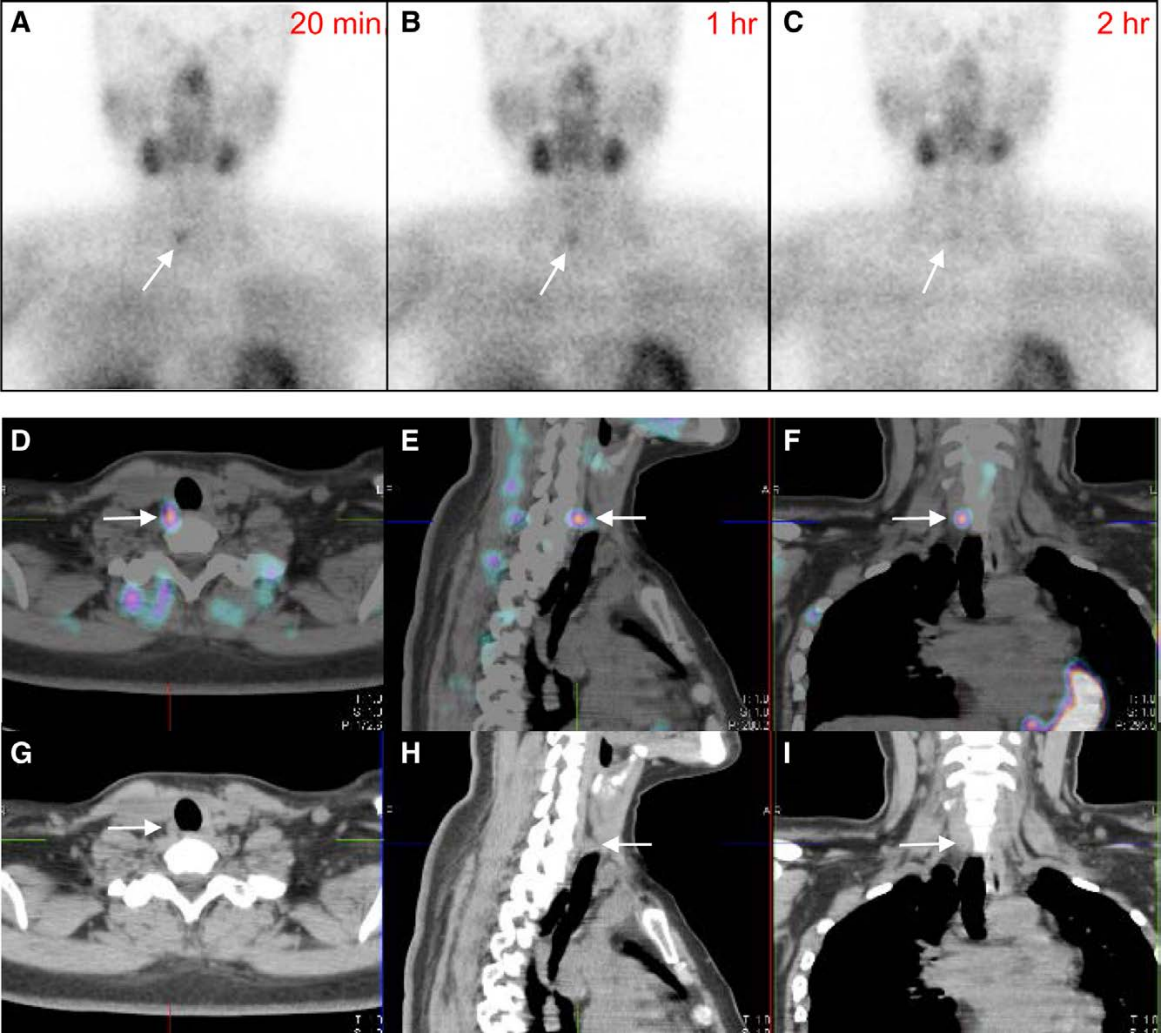
The second Tc-99m MIBI scan and SPECT/CT. Mild focal uptake was observed in the right lower thyroid area in the 20-min image (A). This uptake was sustained on the 1-h and 2-h images (B, C). Additional SPECT/CT revealed a 0.8 × 0.4-cm sized nodular lesion in the lower portion of the right thyroid bed (D–F, fusion images, G–I, CT images). Arrows indicate the lesion.

Since the total calcium and PTH levels remained consistently elevated at 11.9 mg/dL and 110.42 pg/dL, respectively, a Tc-99m MIBI scan was performed again (Fig. 2). The 20-minute image showed mild focal uptake in the right lower thyroid area, which was sustained on the 1-hour and 2-hour images. Further examination with SPECT/CT revealed a 0.8 × 0.4 cm nodular lesion in the lower portion of the right thyroid bed. Parathyroidectomy was conducted for this lesion and parathyroid adenoma was finally confirmed through histologic examination. Thirteen days after the parathyroidectomy, total calcium and PTH levels returned to normal, measuring 8.4 mg/dL and 9.9 pg/mL, respectively. Throughout the 5-year follow-up period, the total calcium and PTH levels remained within the normal range and there were no observations of renal stones or fractures.

At the time of the initial Tc-99m MIBI scan, a dual-energy X-ray absorptiometry (DEXA) scan was performed to assess the patient’s bone mineral density. Z-scores were used considering her premenopausal status. The *Z* scores for her L-spines and hip were −1.6 and 0.3, respectively, indicating that both were within the expected range for her age. The distal third of the radius, which is known to reflect the catabolic effects of parathyroid hormone, was not imaged. In the follow-up DEXA examination conducted 14 months later, the patient’s bone mineral density remained relatively stable with no significant changes.

## 3. Discussion

Tc-99m MIBI is a lipophilic cationic radiopharmaceutical that is taken up by cells through passive diffusion.^[5]^ Mitochondria, which play a crucial role in energy production, have a significant affinity for Tc-99m MIBI.^[5]^ Therefore, Tc-99m MIBI has primarily been utilized for myocardial perfusion assessment, localization of parathyroid adenoma, and breast cancer detection.^[6–9]^ In addition, some studies have reported varying degrees of Tc-99m MIBI uptake in thyroid carcinomas,^[10,11]^ although high uptake is less common compared to breast cancers.

Over the past 2 decades, fluorine-18 fluorodeoxyglucose (F-18 FDG) positron emission tomography/computed tomography (PET/CT) has become a prominent choice for oncology imaging in nuclear medicine. As a consequence, nuclear physicians rarely experience tumor imaging using Tc-99m MIBI. Even in our institute, Tc-99m MIBI is used only for localizing parathyroid adenomas. Although encountering tumor imaging using Tc-99m MIBI is rare, nuclear physicians should be aware that tumors rich in mitochondria, such as thyroid carcinoma, lymphoma, and brain tumors, could exhibit high uptake of Tc-99m MIBI.^[12–14]^

Although there have been reports on patients with concurrent parathyroid adenoma and thyroid carcinoma,^[15,16]^ distinguishing the 2 based on imaging remains challenging. We misdiagnosed thyroid carcinoma as a parathyroid adenoma located within the thyroid gland and missed the presence of a small parathyroid adenoma on the contralateral side. On the contrary, there has been a report of mistaking intrathyroid parathyroid adenoma for thyroid carcinoma.^[17,18]^ Debna et al^[19]^ reported that using multiphase multidetector CT, intrathyroid parathyroid adenomas show higher peak enhancement in the arterial phase and rapid washout compared to colloid nodules and papillary thyroid carcinomas. However, this technique is only feasible in institutes equipped with multiphase multidetector CT and may come with additional costs and radiation exposure.

Approximately 80% of patients with primary hyperparathyroidism are asymptomatic.^[20]^ Our patient also did not show any symptoms when hypercalcemia was detected during a routine health examination. Although the majority of patients who do not meet the criteria for parathyroidectomy experience a benign course with stable bone mineral density and no significant increase in serum calcium and parathyroid hormone, about 25% of patients exhibit evidence of progressive diseases, such as worsening hypercalcemia, hypercalciuria, and reductions in bone mineral density.^[21]^ Therefore, asymptomatic patients should undergo regular monitoring to detect any complications that may necessitate surgery.

This case further emphasizes the clinical utility of SPECT/CT to identify ambiguous lesions seen on 2-dimensional images. The initial SPECT/CT scan clearly revealed a suspicious nodule within the thyroid gland, and the subsequent SPECT/CT scan finally identified the true culprit lesion, the parathyroid adenoma. Im et al^[22^ and Onwanna et al^[23]^ have reported the effectiveness of Tc-99m MIBI parathyroid SPECT/CT as an imaging modality for assessing the functional status of parathyroid adenomas. Based on these reports, we also strongly recommend considering additional SPECT/CT imaging when evaluating cases of parathyroid adenomas.

When a retrospective review of our case and considering characteristics such as the size and calcifications of the left thyroid nodule, the potential for thyroid carcinoma comes back into consideration. If this possibility had been anticipated, the patient could have undergone fine-needle aspiration guided by ultrasonography to confirm thyroid carcinoma, avoiding the need for thyroid lumpectomy and subsequent total thyroidectomy. Such an approach would have facilitated a single surgical procedure of total thyroidectomy.

One crucial point not to be overlooked in this case is the decision made by an endocrinologist and a nuclear physician to conduct a second Tc-99m MIBI SPECT/CT scan when the patient’s total calcium and parathyroid hormone levels did not normalize after total thyroidectomy. It was during this second Tc-99m MIBI SPECT/CT scan that the parathyroid adenoma was finally detected, enabling subsequent parathyroidectomy. This case emphasizes the significance of such interdisciplinary approaches in the diagnosis and treatment of parathyroid adenoma.

This case has several limitations. It represents a rare occurrence where parathyroid adenoma and thyroid cancer coexist, and the accurate diagnosis was achieved through 2 Tc-99m MIBI SPECT/CT scans. Other institutions may face limitations in utilizing SPECT/CT, or they might employ different imaging modalities such as F-18 fluorocholine PET/CT for parathyroid adenoma detection. Therefore, appropriate interpretation and application are warranted based on the resources and capabilities of each institution. Additionally, the uptake of Tc-99m MIBI in parathyroid adenoma and thyroid carcinoma can vary depending on factors such as size, pathology type, and differentiation. Therefore, it should be noted that there may be patients with different presentations compared to the case we presented.

In conclusion, this case shows the incidental detection of a follicular thyroid carcinoma, initially mistaken for a parathyroid adenoma on a Tc-99m MIBI scan in a patient with hypercalcemia. When interpreting Tc-99m MIBI scans, it is essential to keep in mind that various tumors rich in mitochondria, such as thyroid carcinoma, could show high uptake of Tc-99m MIBI.

## Author contributions

**Conceptualization:** Yeon-Hee Han, Seok Tae Lim.

**Formal analysis:** Yeon-Hee Han.

**Funding acquisition:** Yeon-Hee Han.

**Investigation:** Yeon-Hee Han.

**Methodology:** Yeon-Hee Han, Hwan-Jeong Jeong, Sun Young Lee.

**Resources:** Yeon-Hee Han.

**Software:** Yeon-Hee Han, Seok Tae Lim.

**Visualization:** Yeon-Hee Han.

**Writing – original draft:** Yeon-Hee Han, Seok Tae Lim.

**Data curation:** Hwan-Jeong Jeong, Sun Young Lee.

**Writing – review & editing:** Hwan-Jeong Jeong, Sun Young Lee.

**Validation:** Sun Young Lee, Seok Tae Lim.

**Project administration:** Seok Tae Lim.

**Supervision:** Seok Tae Lim.
